# Single-Kidney Transplant on VA-ECMO While Recovering from Post-Heart-Transplant Graft Failure

**DOI:** 10.1155/2018/7431265

**Published:** 2018-06-28

**Authors:** Gabriel Prada, Anjali Agarwal, Jose L. Diaz-Gomez, Robert A. Ratzlaff

**Affiliations:** ^1^Department of Critical Care Medicine, Mayo Clinic, Jacksonville, FL, USA; ^2^Department of Anesthesiology and Perioperative Medicine, Mayo Clinic, Jacksonville, FL, USA

## Abstract

Although no consensus exists on whether extracorporeal membrane oxygenation (ECMO) support is an indication for solid-organ transplantation other than heart and lung, this practice continues to be limited. We present a case of a 55-year-old man who was placed on venoarterial ECMO (VA-ECMO) during orthotopic heart transplantation (OHT) because of acute graft failure. Twenty-four hours later, the patient underwent deceased-donor renal transplantation (DDRT) while on VA-ECMO and had no complications. On post-DDRT day 2 (post-OHT day 3), the patient was successfully decannulated from VA-ECMO and ultimately discharged home. This case highlights the potential successful use of ECMO support during solid-organ transplantation other than heart and lung and the importance of trained providers and tailored anticoagulation. To the authors' knowledge, this is the first report of perioperative ECMO use during kidney transplantation after recent heart transplantation.

## 1. Introduction

Extracorporeal life support (ECLS) or extracorporeal membrane oxygenation (ECMO) is used to support patients with cardiac or respiratory failure, or both, which is refractory to conventional therapy [1]. Indications for ECMO are expanding, and perioperative use of venoarterial (VA) and venovenous (VV) ECMO systems has been implemented increasingly for candidates of heart or lung transplantation as bridge therapy to transplant or for recovery in patients in whom early severe graft failure develops after transplantation [2].

Although no consensus exists on whether ECMO support is an indication for solid-organ transplantation other than heart and lung, this practice continues to be limited. To the authors' knowledge, there are no reports of perioperative ECMO support during kidney transplantation. The authors present the case of a 55-year-old man who was placed on VA-ECMO during heart transplantation because of acute graft failure and then underwent single-kidney transplantation while on VA-ECMO, with no complications.

## 2. Case Report

A 55-year-old Caucasian male was admitted to our hospital with a history of biventricular systolic heart failure due to ischemic cardiomyopathy and chronic stage IV kidney disease due to diabetic nephropathy; one month earlier, he had been approved for combined heart-kidney transplant. He recently had increased fatigue and dyspnea, weight gain of 8 kg, and serum creatinine that increased from 2.3 mg/dL to 3.7 mg/dL despite outpatient inotropic and diuretic therapy. Other comorbidities included antiphospholipid syndrome under warfarin management, multiple myocardial infarctions, with 2 coronary artery bypass grafting procedures, placement of biventricular automated implantable cardioverter-defibrillator, and placement of left ventricular assist device (HeartWare) 5 months earlier.

On hospital day (HD) 14, after optimization of hemodynamics with diuretics and inotropic support, the patient was listed for heart-kidney transplant and transferred to the intensive care unit. On HD 24, the patient was taken to the operating room for combined heart-kidney transplant; however, only orthotopic heart transplantation (OHT) was possible. During the OHT, acute right ventricular graft dysfunction developed, resulting in cardiogenic shock, requiring placement of VA-ECMO support through central cannulation and withholding of the single-kidney transplantation. VA-ECMO was utilized to allow the newly transplanted heart to rest and recover and to optimize hemodynamics and volume status in order for the patient to eventually receive the single-kidney transplantation. Simultaneously, he received support with vasopressin 0.04 U/min, dobutamine 10 mcg/kg/min, epinephrine 0.01 mcg/kg/min, and full-dose anticoagulation with heparin 9.5 U/kg/hour. Tables 1 and 2 show the initial and subsequent laboratory results and ECMO parameters.

On post-OHT day 1, after the patient had improved volume and cardiovascular status, adequate urine output (1,660 mL/24 hours), and stable laboratory testing parameters, the heparin infusion was reduced to 5.5 U/kg/hour (i.e., low dose) and the patient underwent deceased-donor renal transplantation (DDRT). During the DDRT procedure, the patient received 1 unit of packed red blood cells and had no complications. After DDRT, the patient required epinephrine at 0.03 mcg/kg/min, dobutamine at 10 mcg/kg/min, and heparin at 5.5 U/kg/hour.

During the first 48 hours after DDRT, the patient's urine output was adequate (1,900 mL in 24 hours) and his serum creatinine concentration decreased to 2.0 mg/dL. On post-DDRT day 2 (post-OHT day 3), after repeated transesophageal echocardiography showed improved right ventricular function and optimal volume status, the patient underwent ECMO decannulation, chest washout, and sternal closure without complication. His renal function continued to improve, and his anticoagulation was discontinued. The only inotropic support needed was dobutamine at 7.5 mcg/kg/min. On post-DDRT day 5 (post-OHT day 6), the patient was successfully weaned from mechanical ventilation and on post-DDRT day 18 (post-OHT day 19) was transferred to the posttransplant care unit for further care.

## 3. Discussion

The primary indications of ECMO are severe respiratory failure, cardiac failure, or combined cardiopulmonary failure with a high mortality risk, where ECMO provides life-saving cardiopulmonary support for patients who would not otherwise survive with conventional therapy [1]. ECMO is used as a bridge to heart or lung recovery, transplantation, or ventricular assist device implantation. Both VA- and VV-ECMO systems have been increasingly implemented in critical care, cardiothoracic surgery, and cardiac and lung transplant practice as an integral pre-, intra-, and postoperative (i.e., perioperative) instrument [2].

The perioperative use of ECMO in candidates for heart or lung transplantation (i.e., bridge therapy) and those who have early severe graft failure has been well described and is considered the standard of care. However, the use of ECMO for patients undergoing solid-organ transplantation other than heart and lung continues to be limited. Transplant surgeons and intensivists have substantial hesitancy, perhaps due to the required anticoagulation state of patients on ECMO; the considerable frequency of adverse events during ECMO for adults, such as cannula site hemorrhage (VV, 13%; VA, 18%), surgical site hemorrhage (VV, 10%; VA, 20%), renal failure (VV, 9%; VA, 12%), and infection (VV, 17%; VA, 13%); and the delicate hemodynamic status [2].

Few reports have been published about ECMO use for patients with liver or kidney transplantation and fewer about perioperative ECMO in liver, combined heart-liver, and lung-liver transplantation. To the authors' knowledge, this is the first report of perioperative ECMO use during a single-kidney transplant after heart transplantation.

Patients with heart failure have increased prevalence of renal and liver disease, whereby the renal and liver disease worsens heart function even further [3, 4]. Acute or chronic heart diseases trigger several humoral, hormonal, and immune-mediated pathophysiological interactions that can lead to acute or chronic kidney disease, respectively, a concept elucidated under the term “cardiorenal syndrome” [5]. Moreover, renal failure is a common and detrimental manifestation among heart transplant recipients and is an independent predictor of overall mortality [6]. In recent years, multiorgan transplant (heart-liver and heart-kidney) has increased substantially as a therapeutic option, showing improvement in quality of life and survival [7]. Of note, patients with end-stage renal failure who undergo kidney transplant subsequent to heart transplant have better survival than those who do not undergo kidney transplant [8].

For patients with severe liver or renal failure whose best therapeutic option is transplant receipt, the concomitant need for ECMO support represents a challenge for the treating team, in part due to the substantial debate around anticoagulation management on ECMO (either VA or VV). On one hand, continuous contact between blood and foreign surfaces of the ECMO circuit shifts the hemostatic balance to a hypercoagulable state; on the other hand, the most frequent complications related to ECMO are bleeding events. Additionally, there are no randomized control trials that provide evidence or guidance on this issue. However, use of ECMO with low-dose heparinization may be a good strategy because it has been increasingly reported to reduce perioperative morbidity in lung transplant patients [9].

In the present case, the patient presented with an acute decompensation of his chronic heart failure which had caused acute worsening of his chronic kidney disease, that is, cardiorenal syndrome type 1 (“acute cardiorenal syndrome”) [5]. When deciding the appropriate time to proceed with the DDRT and whether to do so while on VA-ECMO, the authors pondered hemodynamics and volume status recovery, urgency for the DDRT, and anticoagulation. Because the patient's hemodynamics and volume status improved rapidly and considerably enough while on VA-ECMO, the authors decided to proceed with the DDRT while on VA-ECMO and to allow the transplanted kidney to contribute in the overall recovery of the patient. Low-dose heparin intravenous drip administration was started before the DDRT and maintained in the postoperative period until ECMO was removed. Renal function was optimal immediately after the DDRT, and cardiac function improved considerably during the next 48 hours after the DDRT, thereby allowing weaning of the patient from the ECMO on the post-DDRT day 2. VA-ECMO was therefore utilized to facilitate optimal hemodynamics for both newly transplanted organs.

A consensus is lacking on whether ECMO support is a contraindication for solid-organ transplantation other than heart and lung. In addition, no evidence shows negative outcomes related to the ECMO system and the anticoagulation status in this patient population. The authors opine that ECMO support is not an absolute contraindication for solid-organ transplantation other than heart and lung. Skilled ECMO management with trained providers, tailored anticoagulation with low-dose heparinization, and health care infrastructure that can help prevent or manage adverse events are measures that ensure successful ECMO use in delicate yet necessary interventions such as solid-organ transplantation other than heart and lung.

This case report does not allow for the conclusion that every patient on ECMO who is also a candidate for solid-organ transplantation other than lung or heart should undergo the transplant while on ECMO. Currently, the authors are unable to prove whether this approach can improve outcomes in a large population of patients; more cases are required to arrive at solid conclusions.

## Figures and Tables

**Table 1:** Laboratory results before and after OHT and DDRT.

**Test**	**Before OHT**	**Post-OHT ** **day 0**	**Pre-DDRT**	**Post-DDRT** **day 0**	**Post-DDRT** **day 1**	**Post-DDRT** **day 2**
Platelets, x10^9^/L	339	98	111	101	80	89
BUN, mg/dL	88	73	78	81	89	106
Creatinine, mg/dL	3.0	2.5	2.5	2.5	2.4	2.0
PT, sec	22	21	25	25	18	14
INR	2.0	1.8	2.3	2.2	1.5	1.1
APTT, sec	64	92	70	47	36	47

APTT, activated partial thromboplastin time; BUN: blood urea nitrogen; DDRT, deceased-donor renal transplant; INR, international normalized ratio; OHT, orthotopic heart transplant; PT, prothrombin time.

**Table 2:** Extracorporeal membrane oxygenation parameters before and after DDRT.

Parameter	Post-OHT day 0	Before DDRT	Post-DDRT day 0	Post-DDRT day 1	Post-DDRT day 2
Flow, L/min	4.0	4.38	4.3	3.9	2.8
FIO_2_	0.60	0.60	0.60	0.60	0.60
PaO_2_, mm Hg	194	99	108	118	120
Sweep gas, L/min	2.0	2.5	3.5	3.0	3.0

DDRT, deceased-donor renal transplant; FIO_2_, fraction of inspired oxygen; OHT, orthotopic heart transplant.

## References

[1] Thomas V., Brogan L. L., Lorusso R., MacLaren G., Giles P. (2017). *Extracorporeal Life Support: The ELSO Red Book*.

[2] Thiagarajan R. R., Barbaro R. P., Rycus P. T. (2017). Extracorporeal Life Support Organization Registry International Report 2016. *ASAIO Journal*.

[3] Silverberg D., Wexler D., Blum M., Schwartz D., Iaina A. (2004). The association between congestive heart failure and chronic renal disease. *Current Opinion in Nephrology and Hypertension*.

[4] Ocel J. J., Edwards W. D., Tazelaar H. D., Petrovic L. M., Edwards B. S., Kamath P. S. (2004). Heart and Liver Disease in 32 Patients Undergoing Biopsy of Both Organs, with Implications for Heart or Liver Transplantation. *Mayo Clinic Proceedings*.

[5] Ronco C., Haapio M., House A. A., Anavekar N., Bellomo R. (2008). Cardiorenal syndrome. *Journal of the American College of Cardiology*.

[6] Lee J. M., Lee S.-A., Cho H.-J. (2013). Impact of perioperative renal dysfunction in heart transplantation: Combined heart and kidney transplantation could help to reduce postoperative mortality. *Annals of Transplantation*.

[7] Praseedom R. K., McNeil K. D., Watson C. J. E. (2001). Combined transplantation of the heart, lung, and liver. *The Lancet*.

[8] Cassuto J. R., Reese P. P., Sonnad S. (2010). Wait list death and survival benefit of kidney transplantation among nonrenal transplant recipients. *American Journal of Transplantation*.

[9] Biscotti M., Yang J., Sonett J., Bacchetta M. (2014). Comparison of extracorporeal membrane oxygenation versus cardiopulmonary bypass for lung transplantation. *The Journal of Thoracic and Cardiovascular Surgery*.

